# Dyeing of Polyester with Disperse Dyes: Part 2. Synthesis and Dyeing Characteristics of Some Azo Disperse Dyes for Polyester Fabrics

**DOI:** 10.3390/molecules21070855

**Published:** 2016-06-29

**Authors:** Alya M. Al-Etaibi, Huda S. Alnassar, Morsy Ahmed El-Apasery

**Affiliations:** 1 Natural Science Department, College of Health Science, Public Authority for Applied Education and Training, Fayha 72853, Kuwait; 2 Department of Laboratories Technology, College of Technological Studies, Public Authority for Applied Education and Training, Fayha 70654, Kuwait; hsalnassar@yahoo.com; 3 Dyeing, Printing and Textile Auxiliaries Department, Textile Research Division, National Research Centre, 33 El Buhouth St., Dokki, Cairo 12311, Egypt; elapaserym@yahoo.com

**Keywords:** polyester fabric, fastness properties, dyeing characteristics, carriers

## Abstract

The goal of this study was to utilize carrier for accelerating the rate of dyeing not only to enhance dyeing of polyester fabrics dyed with disperse dyes **3a**,**b**, but also to save energy. Both the color strength expressed as dye uptake and the fastness properties of the dyed fabrics were evaluated.

## 1. Introduction

Disperse dyes cause environmental concern due to their widespread utility which has been increased in the textile industry since the discovery of synthetic fibers such as polyester. Disperse dyes have particularly low water solubility and to be used from this medium, they must be milled to a very low particle size and dispersed in water using a surfactant (dispersing agent), or else a carrier must be added during dyeing or printing [1,2,3,4]. The real mechanism by which carriers accelerate textile coloration is well known. Polyester fabrics absorb carriers and swell. This swelling can slow down liquor flow in packaging causing lowering of the polymer glass transition temperature (Tg), thus promoting polymer chain movements and creating free volume. This accelerates the distribution of the dye into the fabrics. Alternatively, the carrier may form a film around the surface of the fabrics wherein the dye is soluble, thus increasing the rate of transfer into the fabric [5,6,7].

This paper aims to show the capability of dye adsorption into the fabrics under normal conditions of pressure and temperature in the presence of carrier in an attempt to get high dye uptake; consequently, the amount of dyed, wasted water is reduced and this eliminates hazardous to the environment.

## 2. Results and Discussion

### 2.1. Chemistry

**Scheme 1:**
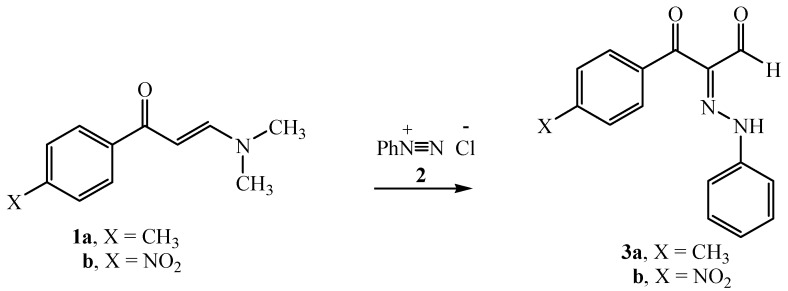
Synthesis of disperse dyes **3a**,**b**.

The synthesis of some dyes based on dimethylamino-*p*-arylpropenones derivatives has reported by us [8]. Herein, in an attempt to estimate their dyeing characteristics, enaminones **1a**,**b** react with phenyl diazonium salts **2** in acidic medium to afford disperse dyes **3a**,**b** (Scheme 1).

Disperse dyes **3a**,**b** were applied to polyester fabrics at 2% shade, without or with using commercial HC carrier at different dyeing temperatures of 70, 90 and 100 °C. Greenish-yellow and yellowish-orange color shades were obtained. The dyeing properties on the polyester fabrics were estimated in terms of their fastness properties (washing, rubbing, light and perspiration). The surface color yield K/S was used to give details on the amount of dye absorbed on the surface of the polyester fabrics. The K/S values outlined in (Table 1 and Table 2) show that dyes **3a**,**b** showed high affinity for the polyester fabrics. 

### 2.2. Effect of Concentration of Carrier

The rate of dye diffusion can be strengthened by increasing the permeability of the fabrics, by increasing the ability of the fabrics to swell. This can be obtained by additional compounds (with smaller molecules than the dye molecules and with definite affinity towards the fabric) in the dye bath. These compounds are named as carriers, as a result of their small molecule size, and they rapidly distribute into the fabrics. The use of carriers is a well-known method for increasing the speed of the distribution of dyes in polyester fabrics. Carriers incite the loosening of the fabric microstructure [9]. Commercial HC was selected as an accelerator or as a carrier to be added to the dye bath to assist the dyeing process, especially if the dyeing process was carried out under the boiling point.

**Figure 1:**
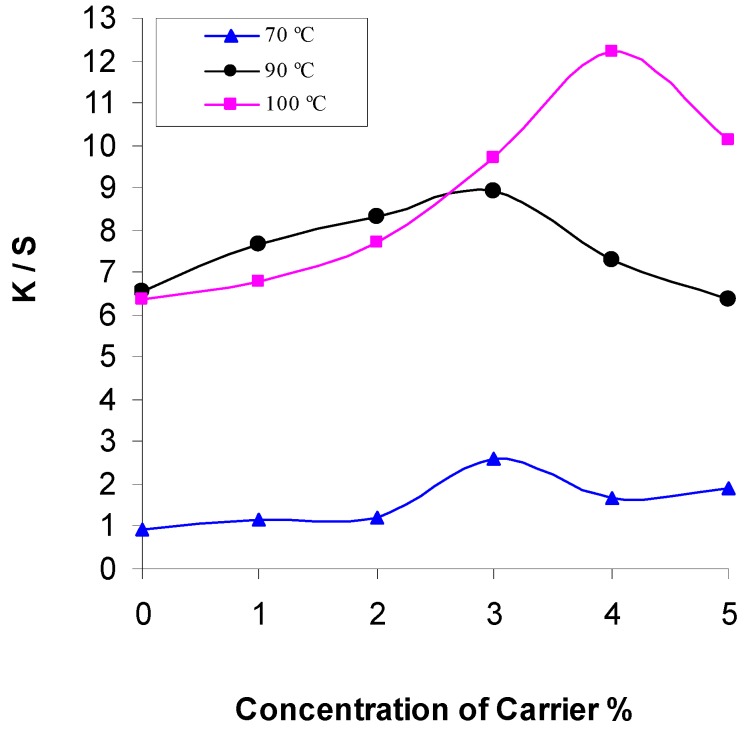
Effect of the concentration of carrier on the dyeing for disperse dye **3a** at different dyeing temperatures.

**Figure 2:**
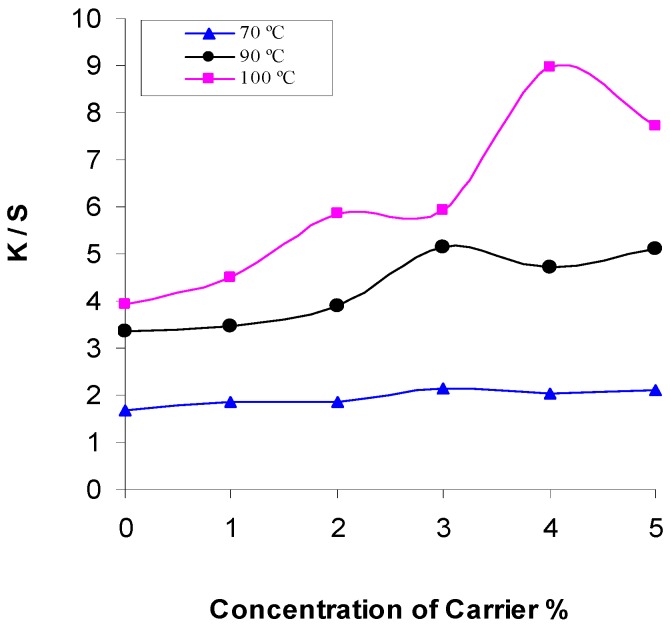
Effect of the concentration of carrier on the dyeing for disperse dye **3b** at different dyeing temperatures.

Figure 1 and Figure 2 show that in the case of dyeing polyester fabrics with disperse dyes **3a**,**b** at 70 and 90 °C, with the increase of the concentrations of the carrier from zero (without using carrier) to 5% of the weight of the fabric, there is a slight increase of color strength K/S values until a 3% concentration of the carrier. In the case of dyeing polyester fabrics with dispersed dyes **3a**,**b** at the boiling temperature (100 °C), the highest color strength K/S value was obtained at a 4% concentration of the carrier. Moreover, the ∆E of both dyes **3a**,**b** increases with the increase of the concentration of the carrier.

It can be concluded from the data in Table 1 that the color strength K/S value of the pretreated fabric with disperse dye **3a** increases by increasing the carrier concentration to 3% and 4% at different dyeing temperatures of 70, 90 and 100 °C. The maximum color strength K/S values (2.60, 8.90 and 12.21) were obtained, since the color strength K/S values are found to increase by about 179%, 36% and 92%, respectively, compared to those of the sample dyed without adding carrier. Also, it can be concluded from the data in Table 2 that color strength K/S values of the pretreated fabric with disperse dye **3b** increase by increasing the carrier concentration to 3% and 4% at different dyeing temperatures of 70, 90 and 100 °C. The maximum color strength K/S values (2.13, 5.15 and 8.97) were obtained, since the color strength K/S values are found to increase by about 26%, 54% and 128%, respectively, compared to those of the sample dyed without adding carrier. Carriers play an important role in dyeing polyester fabrics, especially when the dyeing process is carried out under atmospheric pressure. Commercial HC acts as a carrier or plasticizing agent for polyester fabrics, resulting in increasing the number and size of voids within which the molecules of disperse dye can distribute faster inside the molecular structure of the polyester fabrics.

### 2.3. Effect of Dyeing Temperature

To study the significance of dyeing temperature in the dyeing process, the pretreated samples were dyed at different degrees of temperature (70, 90 and 100 °C); after that, the color strength K/S values of the dyed samples were considered. Figure 1 and Figure 2 show that as the temperature of the dyeing of polyester fabrics with disperse dyes **3a**,**b** increases from 70 to 100 °C, the color strength K/S values increase for both dyes without or with carrier, and this may be attributed to the high distribution rate and the increase of the kinetic energy of the dye molecules [10]. 

Moreover, the ∆E of both dyes **3a**,**b** increases by increasing the dyeing temperatures. It is observed from Table 1 and Table 2 that the K/S values of the pretreated samples dyed with disperse dyes **3a**,**b** increase by raising the dyeing temperature, especially when raising the temperature from 70 to 90 °C, since there was a notable increase in the color strength K/S. 

The increasing percentages in K/S are 242% and 141%, respectively. In the same way, the K/S values of the dyed samples with disperse dyes **3a**,**b** increase, by raising the dyeing temperature from 90 to 100 °C, to 37% and 74%, respectively.

This could be due to the possibility of the existence of dye molecules in an aggregated form at lower temperatures, so the solubility will increase at higher temperatures of dyeing [11]. In addition, the carrier is able to penetrate from boiling solutions inside the fabrics.

Alternatively, the temperature shows a very valuable function in establishing the state of the molecular polymer chains. By raising the temperature, the movement speed of the segment polymer chains is increased, resulting in a decrease in the glass transition temperature (Tg) in addition to the dyeing transition temperature (Td) decrease. Therefore, the number and volume of voids will be greater and the diffusion of disperse dye molecules will be easier and faster; thus, a higher amount of dye inside the fabric and, consequently, a higher color strength are obtained. It is distinguished that the rate of dye uptake, in addition to the total dye uptake, increases with an increase in temperature [12] since the dyeing route is endothermic [13,14]. The fastness ratings are outlined in Table 3, showing that the disperse dyes displayed excellent fastness levels to washing, rubbing and perspiration. The light fastness of the dyes **3a**,**b** displayed fair fastness on polyester fabrics. It is of value to mention here that carrier dyeing improves the washing fastness. Attempts are being undertaken to improve the light fastness of the dyed samples by using zinc oxide nanoparticles, and added value will also be obtained, e.g., self-cleaning, absorbing UV radiation, and antibacterial textiles.

## 3. Materials and Methods

### 3.1. General Procedure for the Synthesis of Disperse Dyes *(**3a**,**b**)*

A cold solution of the diazonium salt (10 mmol), prepared by adding a cold solution of sodium nitrite (0.7 g) in water (10 mL) to a solution of aniline (10 mmol) in conc. hydrochloric acid (4 mL), was added to a cold solution of dimethylamino-*p*-arylpropenones **1a** or **1b** (10 mmol) in ethanol (15 mL) containing sodium hydroxide (1.2 g). The resulting mixture was stirred at room temperature for 30 min. The precipitate that formed was collected by using filtration and crystallized from ethanol. Dyes **3a**,**b** were confirmed by the reported data [8].

*3-Oxo-2-(phenylhydrazono)-3-p-tolyl-propionaldehyde* (**3a**). This compound was obtained as greenish-yellow powder (86%); IR (KBr): = 3130 (NH), 1662, 1635 cm^−1^ (2CO); ^1^H-NMR (DMSO-*d*_6_): δ = 2.41 (s, H, CH_3_), 7.21–7.54 (m, 9H, arom-H), 9.98 (s, 1H, CHO, D_2_O exchangeable), 14.21 (s, 1H, NH, D_2_O exchangeable). MS, *m*/*z* (%), 266 (M^+^, 100), Anal. Calcd. for C_16_H_14_N_2_O_2_; C, 72.16; H, 5.30; N, 10.52. Found: C 71.99; H 5.33; N 10.23.

*3-(4-Nitrophenyl)-3-oxo-2-(phenylhydrazono)-propionaldehyde* (**3b**). This compound was obtained as orange powder (84%); mp. 190 °C IR (KBr): = 3127 (NH), 1660, 1633 cm^−1^ (2CO); ^1^H-NMR (DMSO-*d*_6_): 7.11–7.83 (m, 9H, arom-H), 9.92 (s, 1H, CHO, D_2_O exchangeable), 14.18 (s, 1H, NH, D_2_O exchangeable). MS, *m*/*z* (%), 297 (M^+^, 100), Anal. Calcd. for C_15_H_11_N_3_O_4_; C, 60.61; H, 3.73; N, 14.14. Found: C, 60.61; H, 3.69; N, 14.48.

### 3.2. Fabrics

Scoured and bleached 100% polyester fabric was supplied by El-Mahalla El-Kobra Company, El-Mahalla, Egypt. The fabrics were scoured in aqueous solution having a liquor ratio of 1:50 and containing 2 g/L of nonionic detergent solution (Hostapal; Clariant, Swiss, Switzerland) and 2 g/L of Na_2_CO_3_ at 50 °C for 30 min to remove waxes and impurities, then rinsed thoroughly in cold tap water, and dried at room temperature.

### 3.3. Dyeing Process

Dyes **3a**,**b** were used as pure powder in the same form as prepared without milling. Fabric samples (2 g) were introduced into a flask containing a dyebath of 2% (o.w.f) dye shade and Matexil DA-N (supplied by ICI Company, London, UK) as dispersing agent, commercial HC carrier (Liquid) supplied by Egyptian Turkish Co. (Cairo, Egypt) for auxiliaries with different ratio 0–5% (o.w.f) at different dyeing temperatures 70, 90 and 100 °C with a 1:50 liquor ratio. During dyebath preparation, the dye was mixed with 10 drops of DMF and then mixed with dispersing agent, and water was added to prepare a homogeneous dispersion of the dye. The pH was adjusted to 4.5 by using acetic acid. At the end of the dyeing process after 1 h, the dyed samples were removed, rinsed in warm water, and subjected to reduction clearing in a solution comprising 2 g/L of sodium hydrosulphite and 2 g/L of sodium hydroxide (caustic soda) for 10 min at 60 °C, with a liquor ratio of 1:40, and the reduction-cleared sample was rinsed thoroughly in water. The dyed samples were removed, rinsed in tap water, and allowed to dry in the open air.

### 3.4. Color Measurements

The colorimetric parameters of the dyed polyester fabrics were determined on a reflectance spectrophotometer. The color yields of the dyed samples were determined by using the light reflectance technique performed on an UltraScan PRO D65 UV/VIS Spectrophotometer (HunterLab, Reston, VA, USA). The color strengths, expressed as K/S values, were determined by applying the Kubelka-Munk equation:

(1) K/S = [(1 − R)^2^/2R] − [(1 − R_o_)^2^/2R_o_]

where R is the reflectance of colored samples and K and S are the absorption and scattering coefficients, respectively. R_o_ = decimal fraction of the reflectance of the undyed fabric.

CIE Lab Difference

(2) ∆E = (∆*L*^2^ + ∆*a*^2^ + ∆*b*^2^)^½^

∆E: the total color difference between the sample and the standard: *L* represents the white-black axis, *a* represents the red-green axis and finally *b* represents the yellow-blue axis.

### 3.5. Fastness Properties

Fastness properties of the dyed samples were tested to rubbing, perspiration, washing and light fastness according to AATCC standard tests [15].

## 4. Conclusions

The color strength K/S and ∆E exhibited a significant increasing from using a dyeing method with a commercial HC carrier. The obtained results gave excellent fastness properties to washing, rubbing, perspiration and fair light fastness levels as well. Also, the dyeing process with a carrier for polyester fabrics has great advantages such as saving energy when dyeing at low temperatures with a better dye uptake.

## Figures and Tables

**Table 1:** Optical measurements of disperse dye **3a** on the polyester fabrics.

Dyeing Temp.	% Carrier	K/S	L*	a*	b*	C*	h*	∆E
70 °C	0	0.93	87.83	−3.14	4.06	5.13	127.57	87.98
	1	1.17	87.91	−4.66	8.02	9.28	120.16	88.40
	2	1.19	87.25	−5.73	11.47	12.82	116.56	88.19
	3	2.60	86.26	−8.18	17.57	19.36	114.85	88.40
	4	1.68	87.47	−7.48	16.44	18.06	114.45	89.32
	5	1.92	87.51	−8.59	20.05	21.81	113.20	90.19
90 °C	0	6.53	84.86	−12.67	39.74	41.71	107.68	94.56
	1	7.65	85.08	−13.08	41.58	43.59	107.46	95.60
	2	8.32	84.98	−13.72	43.31	45.43	107.58	96.36
	3	8.90	84.79	−13.42	54.47	47.04	106.44	97.14
	4	7.30	85.31	−13.48	39.82	42.04	108.70	95.10
	5	6.37	85.33	−12.95	83.59	40.70	108.55	94.54
100 °C	0	6.34	84.82	−12.70	83.64	40.68	108.19	94.07
	1	6.80	84.83	−13.26	41.02	43.11	106.91	95.16
	2	7.72	84.95	−13.65	43.28	45.38	107.51	96.31
	3	9.70	84.82	−14.11	48.32	50.34	106.27	98.63
	4	12.21	84.81	−14.26	52.16	54.08	105.29	99.58
	5	10.12	84.75	−14.05	48.40	50.39	106.19	98.44

**Table 2:** Optical measurements of disperse dye **3b** on the polyester fabrics.

Dyeing Temp.	% Carrier	K/S	L*	a*	b*	C*	h*	∆E
70 °C	0	1.69	83.92	−1.84	16.74	16.85	96.28	23.29
	1	1.85	83.08	−1.40	22.01	22.06	93.64	28.54
	2	1.87	84.37	−1.52	12.80	12.89	96.76	19.33
	3	2.13	82.61	−1.08	26.28	26.30	92.35	32.78
	4	2.04	83.25	−2.83	24.52	24.68	96.59	31.13
	5	2.10	82.83	−2.86	27.35	27.50	95.98	33.97
90 °C	0	3.34	80.37	−0.67	39.36	39.38	90.98	46.00
	1	3.47	80.77	−1.64	40.94	40.97	92.29	47.55
	2	3.90	81.26	−3.19	42.35	42.47	94.30	48.99
	3	5.15	80.43	−1.13	43.50	43.51	91.84	50.10
	4	4.73	81.33	−2.42	41.72	41.79	93.32	48.29
	5	5.12	80.06	−2.07	43.69	43.47	92.71	50.40
100 °C	0	3.93	79.81	−0.19	41.51	41.52	90.26	48.20
	1	4.49	80.23	−1.27	44.49	44.50	91.64	51.11
	2	5.85	78.46	−1.12	49.55	49.57	88.71	56.34
	3	5.92	79.28	0.11	46.04	46.06	89.86	52.75
	4	8.97	78.08	−0.21	51.20	51.21	90.23	58.04
	5	7.71	77.87	2.34	50.33	50.38	87.34	57.21

**Table 3:** Fastness properties of azo disperse dyes on polyester fabrics.

Dye No	Washing Fastness			Rubbing Fastness		Perspiration Fastness						Light Fastness
						Alkaline			Acidic			
	Alt	SC	SW	Dry	Wet	Alt	SC	SW	Alt	SC	SW	
**3a**	5	5	5	5	5	5	5	5	5	5	5	3–4
**3b**	5	5	5	5	5	5	5	5	5	5	5	3–4

Alt = alteration; SC = staining on cotton; SW = staining on wool.
